# Physicians' Characteristics Associated with Exploring Suicide Risk among Patients with Depression: A French Panel Survey of General Practitioners

**DOI:** 10.1371/journal.pone.0080797

**Published:** 2013-12-10

**Authors:** Aurélie Bocquier, Elodie Pambrun, Hélène Dumesnil, Patrick Villani, Hélène Verdoux, Pierre Verger

**Affiliations:** 1 INSERM, UMR912 “Economics and Social Sciences Applied to Health & Analysis of Medical Information” (SESSTIM), Marseille, France; 2 Aix Marseille University, UMR_S912, IRD, Marseille, France; 3 ORS PACA, Southeastern Health Regional Observatory, Marseille, France; 4 Bordeaux University, U657, Bordeaux, France; 5 INSERM, U657, Bordeaux, France; University of Rochester, United States of America

## Abstract

**Background:**

General practitioners (GPs) have a key role to play in suicide prevention, but the rates at which they question patients with depression about suicidal thoughts and plans are rather low. Little is known about GPs' characteristics associated with such inquiries. Our objectives were to describe GPs' attitudes, perceived barriers, and self-reported practices in this questioning of these patients and to analyze factors associated with these practices.

**Methodology:**

This cross-sectional survey was conducted among participants in a panel of randomly selected French GPs (1249/1431 participated: 87.3%). GPs were interviewed with a standardized questionnaire covering their professional and personal characteristics, attitudes, and practices in exploring the suicide risk of their patients with depression. We built a suicide inquiry score by summing the responses to 5 items and used a multiple linear regression analysis to explore the characteristics associated with this score.

**Principal Findings:**

Most GPs reported inquiring about the presence of suicidal ideation often or very often; less than 30% reported that they frequently explored signs of a specific suicide plan. The mean suicide inquiry score was 12.4 (SD, 2.9; range, 5–20). False ideas, such as thinking that patients who report suicidal ideas do not often commit suicide, were frequent (42.3%). Previous continuing medical education on suicide, participation in a formal mental health network, and patients who committed suicide in the past 5 years were associated with a higher score. Reluctance to question patients about suicide and perception of insufficient skill were associated with a lower score.

**Conclusions/Significance:**

This study showed great variability in French GPs' practices in exploring suicide risk in patients with depression. Interventions aiming at improving GPs' initial training and continuing medical education in suicide and/or depression, and their collaboration with mental health specialists should be developed, and their impacts assessed.

## Introduction

Suicide is a major public health problem worldwide (accounting for approximately 1 million deaths per year), including in France where the suicide rate (14.7 per 100 000) remains one of the highest in Europe [1]–[3]. The presence of a psychiatric disorder is the most consistently reported risk factor for suicidal behavior; more than half of people who die by suicide had a depressive disorder at the time [4]–[6]. Among people with depression, more than half report suicidal ideation [7], and about 4% die by suicide [8]. General practitioners (GPs) have a key role to play in suicide prevention [6], [9] as they manage most cases of depression [10], [11]. Furthermore, half to two thirds of suicide completers visited their GP in the month before their death [9], [12]. Training GPs to recognize and treat depression appropriately can reduce population rates of completed and attempted suicide [13].

All practice guidelines recommend that persons with depression be screened for suicide risk by exploring their suicidal thoughts and plans [1], [5], [14]–[18]. If suicidal ideation or intentions are detected, then risk factors for suicide and the imminence of suicidal behavior must be assessed [5]. Evidence from the literature shows great variability in primary care physicians' practices in exploring suicide risk [19]–[23]. In postal surveys, about 80% of British GPs stated that they sometimes screened for suicidal ideation among patients with signs or symptoms of depression [19], and 58% of American primary care physicians reported direct questioning about suicide when they managed their most recent case of a patient with depression [20]. Two recent American studies using standardized patient designs found lower rates of suicide risk screening (36–42%) [21], [22]. Other studies have found that GPs detect suicidal ideation in 20% to 50% of patients presenting with such ideation [24]–[26].

Only a few studies have analyzed physicians' characteristics associated with exploring suicide risk [21], [22], [26]; they found that both personal (e.g., age, personal experience with depression) and professional (e.g., participation in continuing medical education, CME) characteristics are linked with this exploration. Potential relations between physicians' attitudes towards suicide (e.g., false ideas about suicide management) and their practices in exploring suicide risk are also assumed [19], [27], [28], but, to the best of our knowledge, have not been explored by a quantitative approach.

This study among a panel of French GPs aimed to i) describe their attitudes towards suicide, perceived barriers, and self-reported practices in questioning patients with depression about suicidal thoughts and plans; and ii) investigate whether GPs' self-reported practices were associated with their attitudes, perceived barriers towards suicide management, or personal and professional characteristics.

## Materials and Methods

### Sampling

In 2008, around 58,000 GPs (31.6% of them women) were in private practice in France [29]. This survey is the third in a series nested in the national panel of French GPs, designed to collect data regularly about their activity and practices. Its methodology has been presented elsewhere [30]–[32]. Briefly, 5170 GPs were randomly selected from the Ministry of Health's exhaustive database of health professionals in France. Sampling was stratified for location of the general practice (urban, suburban, or rural), gender, age (<49, 49–56, >56), and annual workload, defined by number of office consultations and house calls (<2849, 2849–5494, >5494) in 2008. Information on each GP was obtained from the National Health Insurance Fund (NHIF). To limit a selection bias that might result from particular opinions/attitudes, the specific topics to be studied were not mentioned to GPs before they were asked to participate in the panel.

Of the 5170 private GPs initially selected, 4111 (79.5%) could be contacted after a maximum of 10 attempts; 223 were not eligible, as 112 had already retired or planned to within the next year, 39 planned to move their practice within the next year, and 72 practiced exclusively alternative medicine (e.g., homeopathy or acupuncture). Finally, 1431/3888 eligible and contacted GPs (36.8%) agreed to join, i.e., to respond to 5 consecutive surveys during a 30-month period. The GPs who refused to take part did not differ from participants according to practice location, but were more frequently male (p = 0.02), older (p<10^−3^), and had a higher workload in 2008 (p<10^−3^). Lack of time (46.2%) and lack of interest in the panel (15.6%) were the reasons given most frequently for refusal [30].

### Ethics statement

GPs who agreed to participate in the panel sent back a signed written consent to our team. The National Data Protection Authority, responsible for ethical issues and protection of computerized individual data in France, approved the panel and its procedures.

### Procedure and questionnaire

This cross-sectional survey took place during the last quarter of 2011. Professional investigators contacted the panel members and interviewed them with computer-assisted telephone interview (CATI) software. GPs were interviewed with a standardized 40-item questionnaire covering their professional and personal characteristics and their practices and opinions in the area of depression management. These results have been presented in detail elsewhere [31], [32].

The questionnaire included 5 items on self-reported practices in exploring suicide risk, proposed in a random order (listed in Table 1). For each item, participants were asked to report the frequency (never, sometimes, often, very often) at which they asked these questions of patients with depression. These items correspond to a stepwise approach to exploring suicide risk [5], [6]; the last two items are warning signs that the patient has a detailed and specific suicide plan.

**Table 1:** General practitioners' self-reported practices in exploring suicide risk among patients with depression (French nationwide panel of general practitioners, weighted data^a^).

	Never	Sometimes	Often	Very often
Presence of suicidal ideation (n = 1243)	19 (1.5%)	149 (12.0%)	574 (46.2%)	500 (40.2%)
Intent to commit suicide (n = 1244)	51 (4.1%)	294 (23.6%)	533 (42.8%)	367 (29.5%)
Suicide plan (n = 1245)	222 (17.8%)	356 (28.6%)	429 (34.4%)	239 (19.2%)
Setting one's affairs in order (n = 1241)	519 (41.8%)	375 (30.2%)	243 (19.6%)	105 (8.4%)
Having written a letter (n = 1244)	718 (57.7%)	315 (25.3%)	151 (12.1%)	61 (4.9%)

a All numbers have been rounded due to weighting; this explains differences between the row sums and the total numbers.

We collected information on common inaccurate attitudes towards suicide and suicidal patients [33]–[35]: (i) questioning a patient with suicidal ideas can trigger suicidal behavior; (ii) no one can do much for a person who has decided to commit suicide; (iii) patients who report suicidal ideas do not often commit suicide. GPs were asked to report their degree of agreement with each item (strongly disagree, somewhat disagree, somewhat agree, strongly agree); the answers were subsequently categorized into disagree and agree.

We assessed the following perceived barriers to inquiring about suicide and self-assessed effectiveness in depression management: (i) lack of training on this topic; (ii) reluctance to question patients about suicide; (iii) perception of lack of skill in questioning patients about suicide; (iv) lack of time; (v) perception of effectiveness in managing patients with major depression (yes, no).

We collected information on the following personal characteristics of panel members: (i) age (categorized into ≤50 vs. >50 years, based on the median in the sample); (ii) gender; reported personal history of (iii) depression, (iv) antidepressant treatment; (v) psychotherapy; and (vi) reported history of depression in a close friend or family member; and professional characteristics: (i) area of practice (urban, suburban, or rural); (ii) annual workload (number of office consultations and house calls in 2008 categorized into ≤5000 vs. >5000, based on the median in the sample); (iii) reported CME on suicide risk assessment and management (categorized as never, more than 3 years ago, less than 3 years ago); (iv) participation in a formal mental health network (i.e., a network of GPs and mental health specialists financially supported by regional health agencies aimed at promoting access to and coordination of care).

Two items assessed GPs' experience with suicide among their patients: had any patient attempted suicide and had any completed suicide in the past 5 years (yes, no).

Finally, data on their patients' characteristics were extracted from the NHIF databases, and dichotomized according to the median in the sample. We used proportions of patients on their patient list (all patients seen at least once during 2011) with each of the following characteristics: aged 70 years or more; covered by CMUC (Complementary Universal Health Insurance, a program that exempts individuals who have annual incomes below 9000 € from any out-of-pocket costs [36]), an indicator of a very low socioeconomic status or deprived clientele; and with a chronic disease (ALD status: attributed by the NHIF to persons with specific and expensive chronic diseases that make them eligible for 100% reimbursement for treatment). We selected these items because older age, low socioeconomic status, and chronic psychiatric and/or somatic disease are risk factors for suicidal behavior [2], [5].

### Statistical analyses

Because of the differences mentioned above between GPs who participated in the panel and those who refused, we weighted the data to match the sample more closely to the national French GP population for age, gender, location of practice, and 2008 workload [30], [37].

We built a suicide inquiry score by summing the responses to the 5 items listed in Table 1 (never  = 1, sometimes  = 2, often  = 3, very often  = 4; alpha Cronbach  = 0.7).

First, we conducted descriptive analyses of GPs' attitudes, perceived barriers, experience of suicide among their patients, self-reported practices about questioning patients with depression about suicide, and the suicide inquiry score. Second, we conducted bivariate analyses of the relations between GPs' characteristics and the suicide inquiry score with comparisons of means, and t-tests. Third, we developed a multiple linear regression model to test whether this score was associated with GPs' attitudes, perceived barriers, personal and professional characteristics, or experience of suicide among their patients. The variables entered into the model were all selected a priori based on the literature review; to test the robustness of the results we also ran the model again with a backward procedure. We also calculated the variance inflation factors (VIF) to measure collinearity between the independent variables and checked the model assumptions by analyzing residuals. The coefficient of determination, R^2^, and the adjusted R^2^ were calculated to assess the percentage of variance explained by the model, and the global significance of the model was tested with the F-test.

All analyses were based on two-sided p values, with p<0.05 indicating statistical significance. All analyses were conducted with SAS 9.2 statistical software (SAS Institute, Cary, NC).

## Results

### Sample characteristics

Of the 1431 GPs initially included in the panel, 1249 (87.3%) agreed to participate in this survey. Their mean age was 51.4 years (SD, 8.4), 72.9% were men, and 60.5% worked in urban areas. Those who refused to respond to this survey did not differ significantly from participants for gender, age, place of practice, or number of office visits and house calls in 2008.

### Self-reported practices in exploring suicide risk, attitudes, and perceived barriers

Most GPs (81.0%) reported at least one suicide attempt among their patients over the past 5 years, and 43.0% at least one completed suicide. Most (86.4%) reported that when they saw patients with depression, they inquired often or very often about suicidal ideation: 72.3% about intent to commit suicide; 53.6% about a suicide plan; 28.0% about the patient's setting his or her affairs in order; and 17.0% about whether the patient had written a letter (Table 1). The mean suicide inquiry score was 12.4 (SD, 2.9; range, 5–20; 1^st^ quartile, 10; median, 12; 3^rd^ quartile, 14). Its distribution was close to Gaussian (Figure S1).

According to 9.5% of the GPs, questioning a patient with suicidal ideas can trigger suicidal behavior; 30.4% agreed that no one can do much for a person who has decided to commit suicide, and 42.3% that patients who report suicidal ideas do not often commit suicide (Table 2). The perceived barriers to suicide screening most frequently reported by GPs were lack of training in this risk exploration (37.4%), lack of time to perform it (30.2%), and lack of skill in it (26.2%) (Table 2).

**Table 2:** Associations between general practitioners' attitudes, perceived barriers, and effectiveness and the suicide inquiry score: results of bivariate analysis (French nationwide panel of general practitioners, weighted data).

	n	% column	Mean score	(SD)^a^	p^b^
**Attitudes towards suicide**					
Questioning a patient with suicidal ideas can trigger suicide behavior	1206				0.823
Agree	115	9.5%	12.46	(2.97)	
Disagree	1091	90.5%	12.39	(2.86)	
No one can do much for a person who has decided to commit suicide	1211				0.088
Agree	368	30.4%	12.17	(2.87)	
Disagree	843	69.6%	12.48	(2.89)	
Patients who report suicidal ideas do not often commit suicide	1203				0.034
Agree	509	42.3%	12.19	(2.78)	
Disagree	694	57.7%	12.55	(2.94)	
**Perceived barriers, self-efficacy**					
Lack of training	1225				<10^−4^
Yes	458	37.4%	11.84	(2.81)	
No	767	62.6%	12.71	(2.88)	
Reluctance to question patients about suicide	1228				<10^−4^
Yes	113	9.2%	11.03	(2.66)	
No	1115	90.8%	12.53	(2.87)	
Lack of skill in questioning patients about suicide	1225				<10^−4^
Yes	321	26.2%	11.51	(2.58)	
No	904	73.8%	12.67	(2.94)	
Lack of time	1230				0.016
Yes	372	30.2%	12.06	(2.78)	
No	858	69.8%	12.50	(2.93)	
Perception of effectiveness in managing patients with major depression	1206				0.769
Yes	1109	92.0%	12.37	(2.87)	
No	97	8.0%	12.28	(2.97)	

a Standard deviation.

b p value (t-test).

### Factors associated with the suicide inquiry score

In the bivariate analysis, GPs who agreed that patients who report suicidal ideas do not often commit suicide had a lower suicide inquiry score than the others, and all perceived barriers to exploring suicide risk were associated with a lower score (Table 2). The personal and professional characteristics associated with a higher score were: an age of 50 years or younger, a personal history of psychotherapy, previous CME on the topic of suicide, participation in a formal mental health network, and patients who had attempted or completed suicide in the past 5 years (Table 3).

**Table 3:** Associations between general practitioners' personal and professional characteristics, their experience of suicide, and patients' characteristics and the suicide inquiry score: results of bivariate analysis (French nationwide panel of general practitioners, weighted data).

	n	% column	Mean score	(SD)^a^	p^b^
**Personal characteristics**					
Age (years)	1236				0.036
≤50	508	41.1%	12.58	(2.72)	
>50	728	58.9%	12.22	(3.00)	
Gender	1236				0.381
Male	899	72.8%	12.32	(2.82)	
Female	337	27.2%	12.49	(3.06)	
Personal history of depression	1223				0.095
Yes	215	17.6%	12.67	(3.01)	
No	1008	82.4%	12.28	(2.85)	
Personal history of antidepressant treatment	1225				0.248
Yes	138	11.3%	12.63	(2.97)	
No	1087	88.7%	12.32	(2.87)	
Personal history of psychotherapy	1226				0.012
Yes	162	13.2%	12.95	(3.17)	
No	1064	86.8%	12.26	(2.82)	
Reported history of depression in a close friend or family member	1231				0.694
Yes	520	42.3%	12.32	(2.87)	
No	711	57.7%	12.39	(2.90)	
**Professional characteristics**					
Area of practice	1236				
Urban	749	60.6%	12.30	(3.12)	0.297
Suburban or rural	487	39.4%	12.47	(2.58)	
Annual workload (number of office consultations and house calls)	1234				0.801
<5000	650	52.7%	12.35	(3.13)	
≥5000	584	47.3%	12.39	(2.64)	
CME^c^ on suicide	1236				<10^−4^
Less than 3 years ago	215	17.4%	13.54	(2.97)	
More than 3 years ago	508	41.1%	12.46	(2.83)	
Never	514	41.6%	11.79	(2.74)	
Participation in a formal mental health network	1232				<10^−4^
Yes	54	4.4%	14.66	(3.29)	
No	1178	95.6%	12.26	(2.83)	
**Experience with suicide among his/her patients**					
Any patient attempted suicide in the past 5 years	1230				<10^−4^
Yes	996	81.0%	12.58	(2.83)	
No	234	19.0%	11.52	(2.89)	
Any completed suicide in the past 5 years	1224				0.005
Yes	526	43.0%	12.65	(2.84)	
No	698	57.0%	12.17	(2.88)	
**Characteristics of the practice's patients**					
Patients ≥ 70 years-old (%)	1236				0.179
<13.3	360	51.0%	12.48	(2.89)	
≥13.3	605	49.0%	12.25	(2.87)	
Patients of very low socioeconomic status (%)	1236				0.787
<5.1	591	47.8%	12.34	(2.89)	
≥5.1	645	52.2%	12.39	(2.89)	
Patients with a chronic disease (%)	1236				0.776
<25.6	587	47.5%	12.39	(2.89)	
≥25.6	649	52.5%	12.34	(2.89)	

a Standard deviation.

b p value (t-test).

c Continuing medical education.

In the multiple linear regression analysis, previous CME on suicide (especially within the past 3 years), participation in a formal mental health network, and a patient with a completed suicide in the past 5 years were associated with a higher score. Reluctance to question patients about suicide and perceived lack of skill in such questioning were associated with a lower score. The suicide inquiry score did not vary according to GPs' attitudes towards suicide (Table 4). Using a backward selection procedure led to similar results except for GPs' age (β = −0.419, p = 0.013; full results available upon request). Collinerarity between independent variables was very small (VIF<2).

**Table 4:** General practitioners' characteristics associated with the suicide inquiry score: results of multiple linear regression analysis (French nationwide panel of general practitioners, weighted data, n = 1118).

	β	SD^a^	p	VIF^b^
Intercept	13.174	0.498	<10^−4^	
**Personal characteristics**				
Age (>50 vs ≤50 years)	−0.324	0.180	0.071	1.158
Gender (female vs male)	0.199	0.204	0.329	1.215
Personal history of depression (yes vs no)	0.236	0.289	0.415	1.810
Personal history of antidepressant treatment (yes vs no)	0.021	0.337	0.951	1.691
Personal history of psychotherapy (yes vs no)	0.118	0.279	0.672	1.300
Reported history of depression in a close friend or family member (yes vs no)	0.020	0.177	0.909	1.131
**Professional characteristics**				
Area of practice (urban vs suburban/rural)	−0.289	0.183	0.116	1.184
Annual workload (≥5000 vs <5000)	−0.024	0.173	0.888	1.104
CME^c^ on suicide (less than 3 years ago vs never)	1.507	0.243	<10^−4^	1.257
CME^c^ on suicide (more than 3 years ago vs never)	0.430	0.190	0.024	1.300
Participation in a formal mental health network (yes vs no)	1.802	0.425	<10^−4^	1.064
**Experience with suicide among his/her patients**				
Any completed suicide in the past 5 years (yes vs no)	0.482	0.171	0.005	1.061
**Characteristics of the practice's patients**				
Patients ≥ 70 years-old (≥13.3 vs <13.3%)	−0.227	0.195	0.244	1.400
Patients of very low socioeconomic status (≥5.1 vs <5.1%)	−0.024	0.203	0.905	1.513
Patients with a chronic disease (≥25.6 vs <25.6%)	0.061	0.207	0.770	1.585
**Attitudes towards suicide**				
Questioning a patient with suicidal ideas can trigger suicide behavior (agree vs disagree)	0.489	0.296	0.098	1.103
No one can do much for a person who has decided to commit suicide (agree vs disagree)	−0.239	0.192	0.214	1.128
Patients who report suicidal ideas do not often commit suicide (agree vs disagree)	−0.270	0.175	0.124	1.106
**Perceived barriers, self-efficacy**				
Reluctance to question patients about suicide (yes vs no)	−0.775	0.317	0.015	1.170
Perception of lack of skill in questioning patients about suicide (yes vs no)	−0.698	0.213	0.001	1.256
Lack of time (yes vs no)	−0.118	0.192	0.542	1.138
Perception of effectiveness in managing patients with major depression (yes vs no)	−0.326	0.318	0.306	1.038
Global F test	<10^−4^			
R^2^	0.1113			
Adjusted R^2^	0.0932			

a Standard deviation.

b Continuing medical education.

c Variance inflation factor.

## Discussion

In this survey of a national panel of randomly selected GPs, more than 80% reported they had patients who had attempted suicide in the past 5 years, and more than 40%, patients who had completed suicide. While most of them reported asking patients with depression about suicidal ideation often or very often, less than 30% reported frequent exploration of more details, especially signs of a specific suicide plan. To assess GPs' practices in exploring suicide risk, we built a score by summing their reported practices in exploring suicidal ideation, intention to commit suicide, a suicide plan, and whether patients have set their affairs in order or written a letter; this score revealed great variability in GPs' practices. Participation in CME about suicide or in a formal mental health network, and having a patient who completed suicide in the past 5 years were all associated with a higher score. Reluctance to question patients about suicide and perceived lack of skill in this questioning were associated with a lower score.

### Limitations and strengths

These findings must be interpreted in the light of the following methodological limitations. First, although the participation rate in this panel was relatively high for panels of physicians requiring participation in repeated surveys (see for example [38]: response rate  = 19%), nonparticipants were more frequently male, older, and had a higher workload than participants. As none of these factors was independently associated with the suicide inquiry score, this may not have modified this score.

Second, the survey questionnaire explored self-reported practices in exploring suicide risk among patients with depression, which may differ from actual practices. Depression is a well-known risk factor for suicide, and a social desirability bias may have led to an overestimation of these practices. The percentage of GPs who reported asking patients with depression about suicidal ideation often or very often (86.4%) is close to that observed in a previous study using a similar design [19]. However, it was higher than in other studies that used standardized patients or vignettes with open-ended questions (2–40%) [21]–[23]; the latter methods reflect actual physician practice more accurately but they are extremely difficult and expensive to use on a large sample of GPs [39]. However, such a desirability bias is less likely to have modified the direction of the associations found in our regression model.

Third, GPs were asked to report these practices among patients with depression on a frequency scale; thus, their responses may partly reflect the characteristics of the patients in their practice (e.g., frequency of severe depression). Such an effect is probably limited since we found no association between the suicide inquiry score and characteristics of their patients.

Finally, the cross-sectional nature of our study prevents any causal inference. In particular, we cannot rule out that GPs who took a CME course on suicide or participated in a formal network would already have had higher suicide inquiry scores before this CME course or network participation.

One important strength of this study is that it included various types of factors potentially associated with GPs' practices in exploring suicide risk: personal and professional characteristics, as well as attitudes towards suicide and perceived barriers to suicide management. Most previous studies on this topic have included a more limited set of explanatory variables [22], [26].

This study suggests several ways to improve GPs practices in suicide risk exploration among patients with depression. Although it can be argued that GPs do not have time for this, previous studies suggest that French GPs spend more time with patients with mental health problems who require the prescription of medication (25 minutes on average) [40] and that consultation length is associated with screening for suicidal ideation [26].

### Comparison with the literature and interpretation of the findings

The lack of association between GPs' self-reported practices in exploring suicide risk and their gender, age, and self-assessed effectiveness in depression management is in line with most previous published studies [21], [22], [26]. GPs' practices were not related to their personal history of depression or antidepressant/psychotherapeutic treatment, in contrast to a previous study that found a positive association between personal experience with depression and suicide exploration [21].

Concerns about the impact of screening for suicidal ideation on the risk of committing suicide have often been put forward to explain low levels of this screening [19], [28], [41]. To the best of our knowledge, our study was the first to test whether GPs' practices in this regard vary according to their attitudes towards suicide, and we found no significant association. The percentage of French GPs in our study who agreed that questioning a patient with suicide ideas can trigger suicide completion (9.5%) was much lower than in a 2008 British study (25%) [19]. It was also lower among GPs who had attended a CME session on suicide in the past 3 years than among the others (results not shown). GPs' attitudes may have changed in recent years, perhaps in response to increasing participation in CME in this field [31], [42].

Previous CME on suicide (with a higher size effect for recent CME) and participation in a formal mental health network were the two factors most strongly associated with this questioning. These results are strikingly different from those we have found related to other aspects of depression management, such as prescriptions of antidepressants or referral for psychotherapy: the latter were strongly associated with GPs' personal characteristics and attitudes, but independent of CME or network participation [31], [32]. As the literature indicates, CME appears to have a positive impact on asking depressed patients about suicidal thoughts [42]–[44]. Trained GPs may conduct in-depth explorations of suicide risk more frequently, partly because they are less reluctant to question patients about suicide and have more skill and expertise in such questioning. Indeed, reluctance and perceived lack of skill were both associated with a lower suicide inquiry score in our study. Trained GPs may also have more knowledge about planning and structuring follow-up for patients with suicidal ideation, or information about support facilities for such patients [45], [46]. Mental health networks seek to improve information, train members, and facilitate collaboration between GPs, psychiatrists, psychologists, and nurses, in both private practice and hospitals [47]. They are thus applying the principles of collaborative care models [6]. GPs who participate in mental health networks may be more likely to conduct in-depth explorations precisely because they can more easily ask for advice when in difficulty, or refer patients at high risk of suicide to specialists [48].

Finally, we found that GPs' previous experience with completed suicide among their patients was common and associated with in-depth exploration. Using a qualitative design, Davidsen showed that patient suicide has a substantial emotional impact on GPs that lasts many years; GPs were struck by guilt, failure, and self-scrutiny when a patient committed suicide [49]. His qualitative study suggested that GPs' propensity to question patients about suicide risk did not change after the suicide of a patient. Our results, however, suggest that such an event may affect GPs' practices.

### Conclusions

This study showed great variability in the practices of French private GPs in the field of suicide risk exploration. Part of the explanation for these differences may lie in the variability in their skills and expertise in questioning patients about suicide, their training in this field, and their collaboration with mental health specialists. Improving GPs' initial training and CME on depression, suicide risk recognition, and management and promoting collaborative care organizations in this field have been shown to be effective in decreasing suicide rates at the population level [13]. Such interventions should be developed, and their impacts on GPs' practices and suicide rates at the population level should be evaluated.

## Supporting Information

Figure S1 **Number of general practitioners according to the suicide inquiry score (French nationwide panel of general practitioners, weighted data, n = 1237).**
(DOC)
