# Supplementary material for: Physicians' Characteristics Associated with Exploring Suicide Risk among Patients with Depression: A French Panel Survey of General Practitioners
Source: PLoS One. 2013 Dec 10;8(12):e80797. doi: 10.1371/journal.pone.0080797 (PMC3858232; doi:10.1371/journal.pone.0080797)
Supplement: Figure S1 — Number of general practitioners according to the suicide inquiry score (French nationwide panel of general practitioners, weighted data, n = 1237). (DOC) [file pone.0080797.s001.doc]

Suicide inquiry score

**Figure S1. Number of general practitioners according to the suicide inquiry score (French nationwide panel of general practitioners, weighted data, n = 1237)**
